# Math Performance and Sex: The Predictive Capacity of Self-Efficacy, Interest and Motivation for Learning Mathematics

**DOI:** 10.3389/fpsyg.2020.01879

**Published:** 2020-08-07

**Authors:** Ascensión Palomares-Ruiz, Ramón García-Perales

**Affiliations:** Department of Pedagogy, Faculty of Education of Albacete, University of Castilla-La Mancha, Albacete, Spain

**Keywords:** math performance, sex, self-efficacy, interest and motivation for math, primary education

## Abstract

Differences between the sexes in education is something of particular interest in much research. This study sought to investigate the possible differences between the sexes in math performance, and to deeply examine the causal factors for those differences. Beginning from the administration of the BECOMA-On (Online Evaluation Battery of Mathematics Skills) to 3,795 5th year primary students aged 10–11, in 16 Spanish autonomous communities and the 2 autonomous cities of Ceuta and Melilla. The results for each sex were compared to their perceptions of self-efficacy about completing the test items, and with their interest in and motivation for mathematics. Statistically significant differences were seen in the variables examined. The boys were generally more engaged with science and technical subjects. Generalizing from studies such as this aims to more thoroughly explore, and improve this situation.

## Introduction

Inequality between the sexes in education is an enormously important issue for society as a whole, not only for those working in the field. Its importance goes beyond the educational arena and is manifested in power dynamics and decision-making in various contexts. The origins of these inequalities may be multidimensional, such as traits, behaviors, and identities determined by socialization processes (Hadjar et al., 2014; García-Perales, 2016; Ministerio de Educación y Formación Profesional, 2019b) causing internal dissonance, on occasion led by undervaluing talent and potential (Pomar et al., 2009).

In this study, we focus on science and technical disciplines, women are underrepresented in these fields (Lehman et al., 2017; Botella et al., 2019; McCullough, 2020), more specifically on math. We aim to thoroughly examine the reasons for differences between the sexes in this area, looking into possible causal factors. Various studies have concluded that boys get better results in tests of math performance (Bennett, 1997; Furnham et al., 1999; Pasarín et al., 2004; Chan, 2006; Sánchez et al., 2008; Llor et al., 2012; Instituto Nacional de Evaluación Educativa, 2013; Ministerio de Educación y Formación Profesional, 2019b), and that there are differences in scientific and mathematical reasoning that favor boys and men (Fox and Denham, 1974; Beltrán and Pérez, 1994; Pasarín et al., 2004; Barbero et al., 2007; Suberviola, 2012). This leads to the necessary interpretation beyond the educational arena that, as stated in PISA 2018, “there is a deficit of representation of girls among students with the highest levels of performance in science and mathematics may explain, at least in part, the persistent gender gap in science, technology, engineering, and mathematics (STEM) courses, fields which are among the best paid occupations” (Ministerio de Educación y Formación Profesional, 2019b, p. 85).

From early ages, boys and girls receive the same math instruction, allowing them to assimilate fundamental algorithms to structure their math reasoning in order to be able to apply it to everyday problems. Modifying the teaching and learning processes to their potentials, interests, and motivations helps to prevent the frustration, boredom, and even rejection that the subject sometimes causes in students (González, 2019). Teacher training for the prevention of these attitudes is essential (Nortes and Nortes, 2020). The differences between the sexes in this area cannot be explained by the existence of innate differences of ability (Ministerio de Educación y Formación Profesional, 2019b).

A favorable disposition toward learning math is fundamental, and many national and international organizations have stressed the importance of working on these attitudes in the classroom (Unión Europea, 2004; Ministerio de Educación, Cultura y Deporte, 2014; Ministerio de Educación y Formación Profesional, 2019b). This has also been reinforced by various studies (Cueli et al., 2013; Mato et al., 2014), with the teacher having a prominent role as one who understands these internal processes (Muñoz and Mato, 2008; Tourón et al., 2012). According to this variable, PISA 2012, the last version to focus on the specific evaluation of mathematics, concluded that pupils’ interest in learning math was low, they did not enjoy their learning very much. Girls’ progress in this area was hindered by anxiety and a lack of confidence (Instituto Nacional de Evaluación Educativa, 2013), and by the different types of education they received depending on their models of socialization both in and outside the family environment (Ministerio de Educación y Formación Profesional, 2019b). Nonetheless, the gap between boys and girls has narrowed in each edition of PISA in Spain (Ministerio de Educación y Formación Profesional, 2019b). Logically, attention must be paid to the *math affective domain* (Palacios et al., 2014). The analysis of these attitudes is fundamental in more capable students because “weaknesses in attitudes toward the study of math not only affect lower performing students or schools, many students who are relatively high achieving are slowed down by their negative attitudes toward math” (Muñoz and Mato, 2008, p. 224).

Students with greater potential for math show, even from the beginning of their learning, intense activity and commitment to tasks, rapid understanding of concepts and algorithms, high capacity for abstraction, high flexibility of thought, and elevated interest and motivation (Kruteskii, 1976; Benavides, 2008; Reyes-Santander and Karg, 2009). The numbers of detected cases of highly intellectually capable female students is lower than for their male peers. By way of illustration, this situation is clear from the national data for Spanish schoolchildren in school year 2017/8, the most recent year for which official data is available (Ministerio de Educación y Formación Profesional, 2020): Table 1.

**TABLE 1:** Prevalence of highly intellectually capable students in Spain by sex.

Region	Total high capacity	Boys girls			
		*n*	%	*n*	%
Andalucía	14420	8819	61.16	5601	38.84
Aragón	478	368	76.99	110	23.01
Asturias	1087	756	69.55	331	30.45
Baleares	1132	762	67.31	370	32.69
Canarias	2235	1425	63.76	810	36.24
Cantabria	138	104	75.36	34	24.64
Castilla and León	742	564	76.01	178	23.99
Castilla La Mancha	510	370	72.55	140	27.45
Cataluña	2108	1392	66.03	716	33.97
Comunidad Valenciana	1637	1142	69.76	495	30.24
Extremadura	331	248	74.92	83	25.08
Galicia	1833	1223	66.72	610	33.28
Madrid	2371	1621	68.37	750	31.63
Murcia	3755	2341	62.34	1414	37.66
Navarra	410	287	70.00	123	30.00
País Vasco	564	407	72.16	157	27.84
La Rioja	350	245	70.00	105	30.00
Ceuta	9	7	77.78	2	22.22
Melilla	3	3	100.00	0	0.00
Spain	34113	22084	64.74	12029	35.26

*Authors creation using statistics from Ministerio de Educación y Formación Profesional (2020).*

As Table 1 makes clear, of the 34,113 students identified as highly intellectually capable in Spain, 22,084 (64.74%) were boys compared to 12,029 (35.26%) girls. In the autonomous communities, Andalucía was the most equal region with 8,819 (61.6%) boys and 5,601 (38.84%) girls, whereas Aragón exhibited the greatest differences with 368 (76.99%) boys and 110 (23.01%) girls. Despite these differences in numbers between the sexes, there are studies that have shown that there are no differences in high abilities between boys and girls (Organización para la Cooperación y el Desarrollo Económico, 2009; Jiménez et al., 2010; Jiménez and Baeza, 2012). In this regard, Pérez and Díaz (1994, p. 110) noted that “studies that pose the hypothesis of a sex-linked hereditary factor for mathematical or spatial aptitude, or a different lateral specialization in the brain for men and women are not very substantial.” In addition, there are studies which noted that girls suffered from more prejudice in the diagnostic process for giftedness (Kerr, 2000; Landau, 2003; Jiménez, 2014), existing stereotypes that influence their academic and professional choices in education (Bian et al., 2017) and showing higher levels of emotional problems with respect to gifted boys (Huang et al., 2020). Therefore, there is a need to give a high profile to giftedness, regardless of sex (Mandelman et al., 2010; Hernández and Gutiérrez, 2014; Jaime and Gutiérrez, 2017; García-Perales and Almeida, 2019).

In summary, in this study we aimed to show the results achieved by students participating in the BECOMA-On according to sex, relating the scores to students own perceptions of self-efficacy in completing the test battery, and their interest in and motivation for math. This study uses a contextual framework in order to provide guidance for setting the most egalitarian educational policies possible.

## Materials and Methods

This was a ex post facto study which attempted to analyze the relationships between a series of quantitative data.

### Participants

According to the statistics from MEFP for the 2018/19 school year, the time of the study, 51.7% of schoolchildren were boys, and 48.3% were girls. The data for primary schoolchildren was 51.6% boys, and 48.4% girls (Ministerio de Educación y Formación Profesional, 2020).

The main sample for the study was selected from 147 infant and primary schools from 16 autonomous communities, and 2 autonomous cities. The participating schools were both publicly funded and private or independent, and in both urban and rural areas. The sample selection method was not random, each autonomous community selected the participating schools in their areas. The distribution of the study sample according to their sex was as follows: male 2002 or 52.75% and female 1793 or 47.25%.

### Variables

The main variable in the study was math performance via the BECOMA-On test. This construct has its educational basis in the elements or thinking that allow a subject to deal with day-to-day situations, and assessing how that contributes to social and cultural progress. It is also based on subjects’ understanding of how mathematical algorithms work, modifying them to each individual situation.

Two additional variables were considered:

• Self-efficacy in completing the test battery: this was systematized from 0, the lowest value, to 10, the highest value. It is a relative belief in the subjects’ ability to complete the test battery, based on feelings, actions, and thoughts. The following question has been collected from schoolchildren once the battery has been completed: “How did the test go? Mark an option from 0 to 10, with 0 being the lowest score and 10 being the highest. It indicates only one option.”
• Interest and motivation toward mathematics as a subject: categorized from 0 (lowest value) to 10 (highest value). This variable has an impact on the students’ intrinsic motivation for the subject, affecting their effort and involvement, and thus their academic performance. After completing the instrument, students have been asked the following questions: “What is your interest and motivation toward the Mathematics area? Mark an option from 0 to 10, with 0 being the lowest score and 10 being the highest. It indicates only one option.”

### Instrument

BECOMA-On is an instrument own creation for the online evaluation of math performance in 5th-year primary students, aged about 10–11. It has 30 items spread over 7 Evaluation Tests (ET): Mathematical interpretation (ET1, items 1–5), Mental arithmetic (ET2, items 6–11), Geometric properties (ET3, items 12 and 13), Logical numerical series (ET4, items 14–19), Discovering algorithms (ET5, items 20 and 21), Conventional units (ET6, items 22–27), and Logical series of figures (ET7, items 28–30). Each item is scored as 0, 1, or 2, where 0 means incorrect, 1 means partially correct, and 2 means correct. The test scores range from 0 to 60. The reliability index for the original battery was 0.83 and the validity indices were between 0.78 and 0.86.

This instrument seeks to produce both a qualitative and quantitative evaluation of the students’ math performance. Using the results, students are placed in one of 7 hierarchical performance levels with different qualitative characteristics, from poorer to better mastery of the subject. The instrument is applied to class groups online, and takes about 45 min to complete.

### Procedure

The study was carried out during February 2019. The schools selected were charged with administering the instrument, and in particular with explaining each test, demonstrating examples, and monitoring the time. Prior to the application, participating teachers were given specific training in the administration and content of the instrument. All of the variables used in the study were collected at the same time as the application of the test battery. In the analysis of the results, descriptive statistics and the mean comparison *t*-test were mainly used. For the treatment of the results, the SPSS V24 program has been used.

## Results

The results are given below in three sections: the relationship between the BECOMA-On results and sex, the relationship between the BECOMA-On results, sex, and self-efficacy regarding the test battery and the relationship between the BECOMA-On results, sex, and interest and motivation for math.

### Relationship Between the BECOMA-On Results and Sex

The item responses are given below for each item by students’ sex: Table 2.

**TABLE 2:** Frequencies for each item response by sex.

Items	Boys			Girls		
	0	1	2	0	1	2
IT 1	498	356	1148	397	334	1062
IT 2	710	753	539	634	685	474
IT 3	640	492	870	524	462	807
IT 4	301	421	1280	283	346	1164
IT 5	273	415	1314	220	360	1213
IT 6	211	660	1131	196	552	1045
IT 7	223	468	1311	170	335	1288
IT 8	432	520	1050	334	447	1012
IT 9	553	582	867	543	542	708
IT 10	701	720	581	658	634	501
IT 11	1021	465	516	936	394	463
IT 12	527	152	1323	428	101	1264
IT 13	290	254	1458	235	182	1376
IT 14	196	374	1432	160	332	1301
IT 15	514	719	769	567	807	419
IT 16	720	765	517	827	642	324
IT 17	769	740	493	766	738	289
IT 18	442	864	696	513	783	497
IT 19	455	870	677	490	853	450
IT 20	629	409	964	642	334	817
IT 21	977	355	670	930	300	563
IT 22	399	149	1454	463	103	1227
IT 23	695	385	922	548	331	914
IT 24	700	488	814	640	486	667
IT 25	422	550	1030	437	492	864
IT 26	547	905	550	570	750	473
IT 27	746	135	1121	798	119	876
IT 28	1072	756	174	924	718	151
IT 29	588	906	508	515	751	527
IT 30	494	578	930	389	442	962
Total	16745	16206	27109	15737	14355	23698

Table 2 demonstrated differences between the sexes which made it necessary to continue with the analysis to determine statistical significance. For example, item 29 was answered correctly by more girls than boys, despite the participating sample of girls being smaller. We performed a *t* test to examine the results in more depth: Table 3.

**TABLE 3:** *t* test for independent samples by sex.

Items	Boys		Girls		t	df	p	d
	M	SD	M	SD				
IT 1	1.32	0.85	1.37	0.82	1.70	3793	0.089	0.06
IT 2	0.91	0.78	0.91	0.79	–0.15	3793	0.881	0.00
IT 3	1.11	0.86	1.16	0.85	1.55	3793	0.122	0.06
IT 4	1.49	0.74	1.49	0.75	1.00	3793	0.923	0.00
IT 5	1.52	0.72	1.55	0.70	1.46	3793	0.145	0.04
IT 6	1.46	0.68	1.47	0.68	0.63	3793	0.528	0.01
IT 7	1.54	0.69	1.62	0.65	3.68	3793	0.000***	0.12
IT 8	1.31	0.80	1.38	0.78	2.70	3793	0.007**	0.09
IT 9	1.16	0.83	1.09	0.83	–2.41	3793	0.016*	0.08
IT 10	0.94	0.80	0.91	0.80	–1.06	3793	0.288	0.04
IT 11	0.75	0.84	0.74	0.84	–0.42	3793	0.673	0.01
IT 12	1.40	0.88	1.47	0.85	2.44	3793	0.015*	0.08
IT 13	1.58	0.73	1.64	0.70	2.27	3793	0.023*	0.08
IT 14	1.62	0.66	1.64	0.64	0.90	3793	0.369	0.03
IT 15	1.13	0.79	0.92	0.74	–8.43	3793	0.000***	0.27
IT 16	0.90	0.78	0.72	0.75	–7.19	3793	0.000***	0.23
IT 17	0.86	0.78	0.73	0.72	–5.23	3793	0.000***	0.17
IT 18	1.13	0.74	0.99	0.75	–5.59	3793	0.000***	0.19
IT 19	1.11	0.74	0.98	0.72	–5.58	3793	0.000***	0.18
IT 20	1.17	0.88	1.10	0.90	–2.42	3793	0.016*	0.08
IT 21	0.85	0.89	0.80	0.89	–1.77	3793	0.077	0.06
IT 22	1.53	0.81	1.43	0.87	–3.70	3793	0.000***	0.12
IT 23	1.11	0.89	1.20	0.88	3.15	3793	0.002**	0.10
IT 24	1.06	0.87	1.02	0.85	–1.50	3793	0.135	0.05
IT 25	1.30	0.80	1.24	0.82	–2.50	3793	0.012*	0.07
IT 26	1.00	0.74	0.95	0.76	–2.28	3793	0.023*	0.07
IT 27	1.19	0.95	1.04	0.97	–4.63	3793	0.000***	0.16
IT 28	0.55	0.65	0.57	0.64	0.83	3793	0.407	0.03
IT 29	0.96	0.74	1.01	0.76	1.91	3793	0.056	0.07
IT 30	1.22	0.82	1.32	0.81	3.86	3793	0.000***	0.12
Total	35.18	10.08	34.44	9.22	–2.34	3793	0.019*	0.08

*Significant at 5% (p < 0.05). **Significant at 1% (p < 0.01). ***Significant at 0.01% (p < 0.001).

As Table 3 shows, the mean score for the overall test battery was 35.18 (SD = 10.08) for boys. and 34.44 (SD = 9.22) for girls. Comparison testing showed that there were statistically significant differences between the sexes. Boys scored higher than girls in items 9, 15, 16, 17, 18, 19, 20, 22, 25, 26, 27, and the Total score. While girls scored higher in items 7, 8, 12, 13, 23, and 30. It was notable that boys scored higher than girls in 5 of the 6 items in the Logical numerical series tests (items 14–19) and in 4 of the 6 items in the Conventional units test (items 22–27). Whereas girls scored statistically significantly higher in the Geometric properties test (items 12 and 13).

To corroborate these differences between sexes for each of the Evaluation Tests (ET) in which each item is integrated, a *t*-test was carried out with the following results: Table 4.

**TABLE 4:** *t* test for independent samples by sex for each evaluation tests.

Evaluation Tests	Boys		Girls		*t*	df	*p*	*d*
	M	SD	M	SD				
ET1 (IT 1-5)	6.36	2.20	6.48	2.17	1.71	3793	0.087	0.05
ET2 (IT 6-11)	7.16	3.27	7.22	3.22	0.56	3793	0.573	0.02
ET3 (IT 12–13)	2.98	1.25	3.10	1.19	3.06	3793	0.002**	0.10
ET4 (IT 14–19)	6.74	3.08	5.98	2.67	–8.16	3793	0.000***	0.26
ET5 (IT 20–21)	2.01	1.41	1.89	1.41	–2.64	3793	0.008**	0.09
ET6 (IT 22–27)	7.19	2.73	6.87	2.64	–3.63	3793	0.000***	0.12
ET7 (IT 28–30)	2.73	1.54	2.90	1.55	3.31	3793	0.001**	0.11

*Significant at 5% (p < 0.05). **Significant at 1% (p < 0.01). ***Significant at 0.01% (p < 0.001).

In Table 4, statistically significant differences have been observed in several of the evaluation tests. In favor of men, in tests 4 or Logical numerical series, *p* < 0.001, 5 or Discovering algorithms, *p* < 0.01, and 6 or Conventional units, *p* < 0.001. In favor of women, in tests 3 or Geometric properties, *p* < 0.01, and 7 or Logical series of figures, *p* < 0.01.

### Relationship Between the BECOMA-On Results, Sex and Self-Efficacy Regarding Completing the Test Battery

The first step in the analysis of this relationship was to examine the frequencies and corresponding percentages: Table 5.

**TABLE 5:** Self-efficacy in completing the test battery by sex.

Self-efficacy	Boys		Girls		Total	
	*f*	%	*f*	%	*f*	%
0	24	0.63	28	0.74	52	1.37
1	14	0.37	12	0.32	26	0.69
2	16	0.42	28	0.74	44	1.16
3	30	0.79	39	1.03	69	1.82
4	55	1.45	67	1.77	122	3.21
5	150	3.95	190	5.01	340	8.96
6	218	5.74	237	6.25	455	11.99
7	421	11.09	413	10.88	834	21.98
8	477	12.57	430	11.33	907	23.90
9	345	9.09	240	6.32	585	15.42
10	252	6.64	109	2.87	361	9.51
Total	2002	52.75	1793	47.25	3795	100.00

**FIGURE 1:**
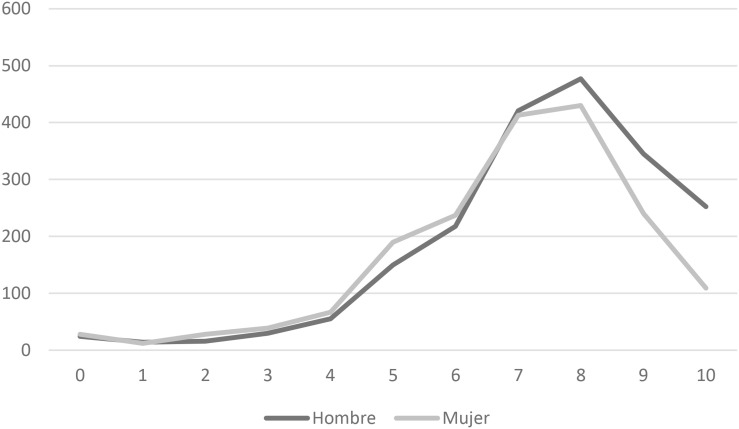
Graphical representation of sex-differences in self-efficacy.

Table 5 shows two notable tendencies. Firstly, girls chose self-efficacy scores of 4, 5, and 6 more than the boys. Secondly, more boys than girls rated themselves with self-efficacy scores of 7, 8, 9, and 10. This is shown graphically below: Figure 1.

These results required deeper analysis to examine possible statistically significant differences. The boys’ mean score was 7.39 (SD = 1.96). and the girls’ was 6.92 (SD = 1.99). The *t* test gave a value of −7.39. *p* < 0.001 and *d* = 0.24. It confirm the existence of statistically significant differences. After continuing to study these differences by means of a regression analysis with a view to establishing the predictive capacity of self-efficacy depending on the sex of schoolchildren, the following results have appeared: Table 6.

**TABLE 6:** Regression analysis between sex and self-efficacy variables.

Model summary				
Model	*R*	*R* square	Adjusted R squared	Std. error of the estimate
1	0.12	0.01	0.01	1.97

ANOVA						

	Model	Sum of squares	df	Mean square	*F*	*p*
1	Regression	212.94	1	212.94	54.67	0.000***
	Residual	14775.11	3793	3.89		
	Total	14988.05	3794			

Coefficients						
	Model	Unstandardized coefficients		Standardized coefficients	*t*	*p*
		B	Std. Error	Beta		
1	(Constant)	6.92	0.05		148.45	0.000***
	Sex	0.47	0.06	0.12	7.39	0.000***

*Significant at 5% (p < 0.05). **Significant at 1% (p < 0.01). ***Significant at 0.01% (p < 0.001).

It has been observed that the contrast made by analysis of variance has given a value of 54.67 (the value of t squared has been 54.61), obtaining a significance of *p* < 0.001. The estimating function of the regression model has been: *Ẏ* = 6.92 + 0.47X. The predicted score for self-efficacy men will be *Ẏ* = 7.39, for women *Ẏ* = 6.92.

### Relationship Between the BECOMA-On Results, Sex, and Interest and Motivation for Math

Table 7 gives the frequencies and percentages for this variable: Table 7.

**TABLE 7:** Interest and motivation for math by sex.

Interest and motivation	Boys		Girls		Total	
	*f*	%	*f*	%	*f*	%
0	53	1.40	33	0.87	86	2.27
1	13	0.34	26	0.69	39	1.03
2	17	0.45	35	0.92	52	1.37
3	46	1.21	36	0.95	82	2.16
4	45	1.19	73	1.92	118	3.11
5	110	2.90	129	3.40	239	6.30
6	137	3.61	160	4.22	297	7.83
7	220	5.80	211	5.56	431	11.36
8	306	8.06	361	9.51	667	17.58
9	380	10.01	364	9.59	744	19.60
10	675	17.79	365	9.62	1.040	27.40
Total	2002	52.75	1793	47.25	3795	100.00

**FIGURE 2:**
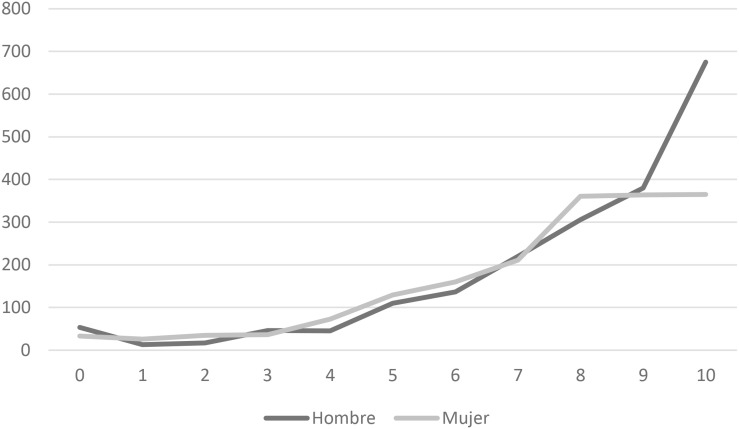
Graphical representation of sex-differences in interest and motivation for math.

Table 7 shows that the results were similar for the options chosen. with the exception of the highest scores. More girls gave themselves a score of 8 than boys, and more boys gave themselves scores of 10. Figure 2 shows this difference graphically: Figure 2.

It was necessary to analyze these results more deeply. The boys’ mean score was 7.94 (SD = 2.38), and the girls’ mean score was 7.47 (SD = 2.37). The *t* test produced an index of −6.09, *p* < 0.001 and *d* = 0.20. It confirm the existence of statistically significant differences. With the aim of deepening these differences, a regression analysis has been carried out to observe the predictive capacity of interest and motivation toward Mathematics according to the sex of schoolchildren, these results have been achieved: Table 8.

**TABLE 8:** Regression analysis between sex and interest and motivation toward Mathematics.

Model summary				
Model	*R*	*R* square	Adjusted R squared	Std. error of the estimate
1	0.10	0.01	0.01	2.37

ANOVA						
	Model	Sum of squares	df	Mean square	*F*	*p*
1	Regression	209.40	1	209.40	37.14	0.000***
	Residual	21386.22	3793	5.64		
	Total	21595.62	3794			

Coefficients						
	Model	Unstandardized coefficients		Standardized coefficients	*t*	*p*
		B	Std. Error	Beta		
1	(Constant)	7.47	0.06		133.19	0.000***
	Sex	0.47	0.08	0.10	6.09	0.000***

*Significant at 5% (p < 0.05). **Significant at 1% (p < 0.01). ***Significant at 0.01% (p < 0.001).

This table has reflected the contrast made through analysis of variance with a value of 37.14 (the value of t squared has been 37.10), reaching a significance of *p* < 0.001. The estimating function of the regression model has been: *Ẏ* = 7.47 + 0.47X. The expected score for men in interest and motivation will be *Ẏ* = 7.94, for women *Ẏ* = 7.47.

## Discussion

One of the basic premises of educational systems is the empowerment of human capital. The integrated development of each person encourages a country’s social, economic and cultural progress. Compulsory schooling is a key time to make the school population participants in learning. Co-education is key work in schools, all students must have the same teaching and learning processes in equal conditions and with equal opportunities, avoiding the discrimination that occasionally coexists with the attempt to offer quality education (Gallardo-López et al., 2020). Differences between sexes may be avoided if suitable measures are put in place (Ministerio de Educación y Formación Profesional, 2019b). In this regard, educational tasks in the curriculum based on scientific-technical skills, such as STEAM (Science, Technology, Engineering, Art and Mathematics). May be an interesting option for encouraging equality between the sexes in the classroom (Crespo-García, 2019).

This is justified by the results from our study. There were statistically significant differences between the sexes in the participating sample. In almost all of the items of the Logical numerical series, and Conventional units sub-testes the boys exhibited higher scores. In contrast, girls scored higher in the items making up the Geometric properties test. In the analysis of the results according to each evaluation test, there are also differences between the sexes, with better results being observed for men in tests with arithmetic content and management of units of measurement, and for women in tests with geometric content. These effects may indicate that sex differences, noted in various studies, may be more linked to results in specific sub-areas of math.

In addition, when comparing the results from boys and girls regarding self-efficacy about completing the test battery, there were statistically significant (*p* < 0.001) differences. Girls selected options 4–6 in the self-efficacy scale more than boys, and boys selected the higher score, 7–10, more than girls. The estimating function of the regression model has been: *Ẏ* = 6.92 + 0.47X. This is despite there being items in which each sex scored higher than the other. In education, beliefs of self-efficacy affect motivation, persistence, and school success (Zalazar et al., 2011; Rosário et al., 2012; Huéscar-Hernández et al., 2020), and there are also social factors (Ruiz, 2005).

The last variable analyzed was students’ interest and motivation for math according to sex. The results were similar between the two sexes, except for the fact that more girls gave themselves scores of 8 than boys, and more boys gave themselves scores of 10 than girls, both statistically significant differences (*p* < 0.001). This was despite some girls outperforming boys, and some boys outperforming girls. The estimating function of the regression model has been: *Ẏ* = 7.47 + 0.47X. These results are in line with findings from other studies (Bazán and Aparicio, 2006; Molera, 2012; Instituto Nacional de Evaluación Educativa, 2013; Mato et al., 2014; Ministerio de Educación y Formación Profesional, 2019b). In addition, this issue may be affected by changes in educational stages, with the transition between primary and secondary school, when attitudes toward math increasingly decline (Mato et al., 2014) and there is the appearance of a lack of interest, motivation, value and faith in ones’ own abilities (Mato, 2010).

Learning math makes educational sense when the knowledge learned in this area is used in the schoolchild’s normal surroundings. In this regard, the value placed on math by the individual, and attitudinal factors are central in learning math. Pleasure in learning math leads to enjoyment for the learner, they perform well, and they find the content interesting (Tourón et al., 2012), as well as exhibiting better attitudes toward homework (Pan et al., 2013). In this way, educational practice must insist on the importance of these considerations or attitudes toward math, and be aware that occasionally the difference between the sexes can come from a discrepancy between what one does and what one could do, from the predisposition to learning rather than problems of attitude. It is unreasonable for the sex of schoolchildren to be a barrier to academic success in this subject. For the development of future research on sex using evaluation tests such as the one presented in this study, other variables could be included for a more exhaustive analysis such as, for example, academic performance, interest and motivation of the scholar toward Mathematics from the point of teaching view and / or academic self-concept. The number of questions for the collection of data related to the variables self-efficacy and interest and motivation in Mathematics will also be increased, with the aim of calculating internal consistency indices of the measurements made. We highlight some data to consider; 75.0% of undergraduates and 71.8% of graduates in Engineering and Architecture are men (Ministerio de Ciencia, Innovación y Universidades, 2019). In 2030 it is expected that 32% of women will reach tertiary education, and 27% of men. Currently the number of women aged 25–64 who have higher education qualifications is 15% higher than the number of men who have those qualifications in Spain (Ministerio de Educación y Formación Profesional, 2019a). Logically, the achievement of personal, social and school wellbeing is essential in educational processes.

## Data Availability Statement

The raw data supporting the conclusions of this article will be made available by the authors, without undue reservation.

## Ethics Statement

The studies involving human participants were reviewed and approved by National Institute of Educational Technologies and Teacher Training (INTEF), the National University of Distance Education (UNED), and the University of Castilla-La Mancha (UCLM). Written informed consent to participate in this study was provided by the participants’ legal guardian/next of kin.

## Author Contributions

RG-P designed the study, collected and analyzed the data, and wrote the manuscript. AP-R contributed to the interpretation of the data and wrote, revised, and refined the manuscript. RG-P and AP-R participated in sending the article to the journal. Both authors contributed to the article and approved the submitted version.

## Conflict of Interest

The authors declare that the research was conducted in the absence of any commercial or financial relationships that could be construed as a potential conflict of interest.
